# APE1/Ref‐1 inhibition via APX3330 lowers monocyte/macrophage infiltration without ameliorating the structure and function of dystrophic *mdx* hindlimb muscles

**DOI:** 10.14814/phy2.70494

**Published:** 2025-08-01

**Authors:** Hannah Lalunio, Craig A. Goodman, Nicole Stupka, Nicholas Giourmas, Danielle A. Debruin, Lauren Sahakian, Kulmira Nurgali, Alan Hayes

**Affiliations:** ^1^ Department of Medicine—Western Health, Melbourne Medical School The University of Melbourne Melbourne Victoria Australia; ^2^ Australian Institute for Musculoskeletal Science (AIMSS) Victoria University Melbourne Victoria Australia; ^3^ Institute of Health and Sport (IHeS) Victoria University Melbourne Victoria Australia; ^4^ Centre for Muscle Research The University of Melbourne Melbourne Victoria Australia

**Keywords:** inflammation, *mdx* mouse, muscular dystrophy, oxidative stress, skeletal muscle

## Abstract

Chronic inflammation and oxidative stress exacerbate muscle wasting and weakness in Duchenne muscular dystrophy (DMD). Apurinic/apyrimidinic endonuclease 1/redox factor‐1 (APE1/Ref‐1) regulates transcription factors involved in inflammatory and oxidative stress pathways. APE1/Ref‐1 is an emerging therapeutic target in inflammatory conditions. This study aimed to investigate the effects of APX3330, a small molecule inhibitor of APE1's Ref‐1 on *mdx* mouse pathology, a model of DMD. Six‐week‐old *mdx* mice and wild type (WT) C57Bl/10 mice were treated with APX3330 (25 mg·kg^−1^) or vehicle for 6 weeks. Ex vivo contractile function, histological and biochemical analysis were performed in extensor digitorum longus (EDL) and soleus muscles. APE1/Ref‐1 protein was greater in *mdx* hindlimb muscles compared to WT (*p* < 0.0001) and APE1/Ref‐1 protein abundance was not altered by treatment with APX3330. In dystrophic EDL muscles, APX3330 treated mice had fewer (47%) infiltrating CD68‐positive monocytes/macrophages (*p* < 0.05) compared to vehicle‐treated *mdx* mice. Markers of oxidative stress, NRF2/KEAP‐1, were unchanged, yet phospho‐NF‐κB abundance was higher with treatment (*p* < 0.01). APX3330 treatment neither improve force output and fatiguability of isolated hindlimb muscles, nor affect muscle pathology. As APE1/Ref‐1 inhibition modestly lowered inflammation, with no improved contractile function, targeting solely inflammation and oxidative stress in 6‐week‐old *mdx* mice appears insufficient.

## 1 INTRODUCTION

One of the most prevalent and severe forms of muscular dystrophy is Duchenne muscular dystrophy (DMD), affecting 1 in every 5000 boys (Aartsma‐Rus et al., 2016). DMD is caused by the loss of the cytoskeletal protein, dystrophin, due to mutations in the *DMD* gene (Hoffman et al., 1987). The absence of dystrophin disrupts the dystrophin‐glycoprotein complex (DGC) and destabilizes the sarcolemma, making muscle fibers highly susceptible to mechanical stress. This results in continuous cycles of damage and insufficient repair, eventually leading to the replacement of contractile tissue with a fatty, fibrotic infiltrate (Desguerre et al., 2009; Li et al., 2015). Heightened inflammation and oxidative stress are interconnected drivers of impaired contractile function, muscle degeneration, and failed regeneration in dystrophic muscle (Dadgar et al., 2014; Haycock et al., 1996; Rodriguez & Tarnopolsky, 2003; Rosenberg et al., 2015). The standard of care for patients with DMD is corticosteroids (prednisolone and deflazacort). These are potent anti‐inflammatory drugs that moderately improve muscle function by delaying the loss of ambulation and improving respiratory capacity (Mah, 2018). However, corticosteroids have adverse side effects, including bone loss, weight gain, cataracts, and diabetes, which limit their effectiveness (Buchman, 2001). Therefore, there is an unmet need for therapies that ameliorate inflammation and improve dystrophic muscle contractile function and pathology, with a more favorable side‐effect profile.

Apurinic/apyrimidinic endonuclease 1/redox factor 1 (APE1/Ref‐1) is a multifunctional protein. The C‐terminus of APE1/Ref‐1 plays a role in the base excision repair (BER) pathway, contributing to genomic maintenance by targeting DNA lesions that increase oxidative stress (Fung & Demple, 2005), while the N‐terminus of APE1/Ref‐1 is involved in redox regulation of transcription factors, including nuclear factor kappa‐light‐chain‐enhancer of activated B cells (NF‐κB), activator protein 1 (AP‐1), signal transducer and activator of transcription 3 (STAT3), and hypoxia‐inducible factor 1‐alpha (HIF‐1α) (Gray et al., 2005; Huang et al., 1996; Nishi et al., 2002; Xanthoudakis & Curran, 1992). APE1/Ref‐1 reduces transcription factors to promote DNA binding and subsequent activation of genes implicated in pro‐inflammatory and oxidative stress signaling cascades (Hirota et al., 1997; Nishi et al., 2002). Conversely, APE1/Ref‐1 repression induces protective, antioxidant effects by enhancing the activity and expression of nuclear factor erythroid 2‐related factor 2 (NRF2) target proteins (Fishel et al., 2015).

To date, the effects of inhibiting the redox domain of APE1/Ref‐1 on inflammation and oxidative stress have been shown to be dependent upon biological context. For example, in vitro studies have reported that overexpressing APE1/Ref‐1 can be protective (Angkeow et al., 2002; Baek et al., 2016; Hao et al., 2019; Kim et al., 2006; Tang et al., 2019); however, this has generally been in acute models of inflammation. In conditions of chronic inflammation and oxidative stress, the repetitive activation of APE1/Ref‐1 may lead to a pathological response. Indeed, APE1/Ref‐1 is elevated in conditions characterized by heightened inflammation and oxidative stress, including chronic inflammatory diseases, such as myocarditis and chronic colitis (Hofseth et al., 2003; Jin et al., 2017; Shin et al., 2015). The small molecule inhibitor, APX3330, which targets the redox domain of APE1/Ref‐1 (Hartman et al., 2021), causes APE1/Ref‐1 to unfold, leading to oxidation of the active disulphide residues, thus rendering the protein unable to reduce transcription factors, keeping them in an oxidized and inactive state (Hartman et al., 2021). This would, in turn, inhibit downstream activity of these transcription factors, including the expression of factors involved in the activation of inflammation and oxidative stress pathways. Indeed, APX3330 has been shown to decrease inflammatory cytokine expression in RAW264.7 cells (Jedinak et al., 2011). Additionally, in the Winnie mouse model of chronic colitis, APX3330 reduced the infiltration of CD45‐positive immune cells in the colon (Sahakian et al., 2020) and reduced mitochondrial superoxide production in the myenteric plexus, indicating that APX3330 alleviated the inflammation‐induced oxidative stress (Sahakian et al., 2020). This reduction in inflammatory and oxidative stress was neuroprotective in enteric neurons, reduced disease severity, and restored GI function (Sahakian et al., 2020). The significant reduction in inflammation and oxidative stress in these models indicates a possible application in other disease states with similar pathology, such as muscular dystrophy (reviewed in [Lalunio et al., 2025]).

To date, few studies have evaluated the role of APE1/Ref‐1 in skeletal muscle (Buck & Chojkier, 1996; Szczesny et al., 2010; Wang et al., 2016; Yuzefovych et al., 2013). As chronic inflammation and oxidative stress are hallmarks of DMD pathology, we hypothesized that APE1/Ref‐1 protein would be upregulated in dystrophic muscles and that APE1/Ref‐1 inhibition with APX3330 treatment would ameliorate dystrophic hindlimb muscle pathology and improve contractile function in C57Bl/10 *mdx* mice.

## 2 METHODS

### 2.1 Animal study

To justify the potential use of APX3330 to inhibit APE1/Ref‐1, it was first necessary to determine if the APE1/Ref‐1 protein is expressed in skeletal muscle. Frozen untreated EDL and soleus muscles from a previous study involving age‐matched (12‐week‐old) wild type (C57Bl/10ScSn) and *mdx* (C57Bl/10ScSn *mdx*) mice (Debruin et al., 2019) were homogenized, and APE1/Ref‐1 protein expression was measured as described below.

Subsequently, we investigated the effect of APE1/Ref‐1 inhibition on dystrophic muscle pathology. Dystrophic (*mdx*) mice and age‐matched wild type (C57Bl/10) mice were kept in a temperature controlled (21°C) environment with a 12:12 h day/night cycle with access to food (SF00‐100, Specialty Feeds) and water ad libitum. At 6 weeks of age, wild type and *mdx* mice were randomly allocated into treatment or vehicle control groups (*n* = 8 per group). Mice either received an intraperitoneal (IP) injection of APX3330 (25 mg·kg^−1^) dissolved in Cremophor (2%), ethanol (2%), and sterile water (96%), or vehicle of only Cremophor (2%), ethanol (2%), and sterile water (96%) twice a day (b.i.d.) for 6 weeks. APX3330 was a generous gift from Prof. Mark R Kelley (Indiana University School of Medicine, Indianapolis, IN). In mice, APX3330 is non‐toxic for doses up to 75 mg·kg^−1^ and has a half‐life of 5.6 h (Kelley et al., 2011). We utilized the same treatment regime used by Fishel et al. (2011) and Sahakian et al. (2020) where, in models of pancreatic cancer and ulcerative colitis, APX3330 lowered inflammatory cell infiltration and the activation of inflammatory cell signaling cascades, including NF‐κB (Fishel et al., 2011; Sahakian et al., 2020).

### 2.2 Tissue extraction

Mice were anesthetized using 4% isoflurane in an induction chamber and maintained on 2% isoflurane through a nose cone with oxygen set to a flow rate of 0.6 mL·min^−1^. Both tendons of the EDL and soleus muscles of the left leg were tied with a 4.0 suture thread and excised for contractile function analysis. Following ex vivo contractile function testing, muscles were blotted dry, tendons removed, weighed, and embedded with optimal cutting temperature (OCT) compound (Tissue‐Tek; Sakura, Torrence, CA, USA) in isopentane precooled in liquid nitrogen for later histological and immunohistochemical analysis. The EDL and soleus muscles of the right leg were collected, weighed, and snap frozen in liquid nitrogen for western blot analysis.

### 2.3 Contractile function testing

Ex vivo evaluation of muscle contractile properties was performed as previously described (Debruin et al., 2020). Briefly, excised EDL and soleus muscles were placed into a contractile chamber (Danish Myo Technology (DMT) A/S, Hinnerup, Denmark) containing Krebs–Henseleit Ringer's solution (118 mM NaCl; 4.75 mM KCl; 1 mM Na_2_HPO_4_; 1.18 mM MgSO_4_·7H_2_O; 2.5 mM CaCl_2_; 24.8 mM NaHCO_3_; 11 mM D‐Glucose; and pH 7.4). Individual baths were bubbled with carbogen (5% CO_2_ in O_2_; BOC gases, Melbourne, Australia) and maintained at 30°C. One end of the muscle was hooked onto a previously calibrated force transducer, and the other was fixed to a micromanipulator with stimulating electrodes positioned to flank the belly of the muscle. Prior to contractility experiments, optimal length (*L*_0_) for each muscle was determined by conducting a series of twitch contractions at increasing lengths to ensure optimal overlap of the sarcomeres, then recorded using calipers.

A force–frequency curve was performed to determine peak force production of each muscle. Both muscles were stimulated at increasing frequencies (10, 20, 30, 40, 50, 60, 80, 100, 120, 150, and 180 Hz) for a duration of 350 ms for the EDL and 500 ms for the soleus, with 3‐min rest periods between pulses to prevent fatigue. Peak tetanic absolute force (P_0_) was recorded as the highest force obtained in the force–frequency protocol, with lower forces recorded as a percentage of the P_0_. Specific force (sP_0_) was determined by normalizing P_0_ to muscle cross‐sectional area (CSA) using the formula CSA = muscle mass/(optimal length × (fiber length/muscle length) × density), where the fiber length to muscle length ratio was assumed to be 0.44 for the EDL and 0.71 for the soleus, and density is 1.06 g/cm^3^ (Brooks & Faulkner, 1988; Close, 1972). Muscle fatiguability was also investigated by exposing the muscles to repeated intermittent electrical stimuli for 3 min, resulting in a marked reduction in force production. The fast‐twitch EDL was stimulated every 4 s at 100 Hz for 350 ms and the slow‐twitch soleus every 2 s at 80 Hz for 500 ms to induce comparable levels of fatigue. Data were collected and analyzed using LabChart Pro version 8 software (ADInstruments, Dunedin, New Zealand).

### 2.4 Histology

#### 2.4.1 Hematoxylin and eosin (H&E)

To assess muscle morphology including damaged areas and regenerating fibers, 10 μM cryosections from EDL and soleus muscles were stained with hematoxylin and eosin (H&E) (Debruin et al., 2020). Images of whole muscle sections at 200× magnification were captured with a Zeiss Axio Imager Microscope (Zeiss, Germany), running the Metafer 4 V3.13.3 imaging software (MetaSystems, Germany) and stitched together by a VSlide V1.1.120 software (MetaSystems, Germany). All images were analyzed using ImageJ software (NIH, Bethsda, MD, USA) with approximately 200 fibers analyzed per sample. A quantitative estimate of degeneration within each section was determined using methods described by Grounds (2014). Specifically, centrally nucleated fibers were counted to determine the percentage of regenerating fibers; necrotic fibers identified by infiltrating inflammatory cells were classified as damaged area to indicate degeneration; and all fibers were individually measured to determine mean fiber size and distribution of fiber size within the muscle cross‐section.

#### 2.4.2 Succinate dehydrogenase (SDH)

To assess changes in oxidative capacity, a succinate dehydrogenase (SDH) staining was performed on freshly cryosectioned EDL and soleus muscle samples (10 μM thick). Briefly, slides were incubated in working solution (0.05% nitro blue tetrazolium; 0.2 M sodium succinate; 0.2 M phosphate buffer, pH 7.6) for 1 h at 37°C, fixed in formal saline (0.9% NaCl, 10% formaldehyde) and mounted in glycerol jelly (Debruin et al., 2020). Images of whole muscle sections at 200× magnification were captured with a Zeiss Axio Imager Microscope (Zeiss, Germany), running the Metafer 4 V3.13.3 imaging software (MetaSystems, Germany) and stitched together by a VSlide V1.1.120 software (MetaSystems, Germany). Images were converted to 8 bit, threshold adjusted to exclude non‐specific staining, and SDH intensity was measured in full cross sections of the muscle (Debruin et al., 2020).

### 2.5 Immunohistochemistry for CD68 and CD45‐positive cells

Immunohistochemical analysis was performed as previously described (McRae et al., 2020). Briefly, cryosections (10 μM) were fixed in 4% paraformaldehyde (PFA) for 10 min and permeabilized in 0.05% TBS‐T (Tris‐buffered saline with 0.05% Tween‐20). Slides were then incubated in a serum‐free blocking solution (Dako, #X0909) for 30 min before a 75‐min incubation in the appropriate primary antibody in antibody diluent (Dako, #S3022) at room temperature. An anti‐CD68 antibody (ab125212, Abcam, 1:500) was used for the detection of monocyte and macrophage infiltration, and an anti‐CD45 antibody (ab40763, Abcam, 1:500) was used to detect leukocyte infiltration. In skeletal muscle, CD45 shows a high level of immunoreactivity with T cells and B cells (Ledbetter et al., 1988). Slides were then incubated with fluorophore‐conjugated secondary Alexa Fluor 594 goat anti‐rabbit antibody (A11012, Thermo Fisher Scientific, 1:1000 in antibody diluent) for 75 min, counterstained with wheat germ agglutinin (WGA; W11262, Thermo Fisher Scientific, 1:50 in PBS), and mounted in mounting medium with DAPI (ab104139, Abcam). Additional sections from untreated *mdx* mice stained without the primary antibody were used as negative controls. Images were captured using a fluorescence microscope at 400× magnification (BX53 upright fluorescence microscope, Olympus). Three images per muscle sample, taken at the top, middle, and bottom of each section, were analyzed using ImageJ software (NIH, Bethsda, MD, USA) and results are expressed as cells/mm^2^.

### 2.6 Western blots

Frozen muscle tissues were homogenized with a handheld Omni homogenizer (Model #TH220) in ice‐cold homogenizing buffer (40 mM Tris–HCl; 1 mM EDTA, pH 8.5; 5 mM EGTA, pH 8.8; 0.5% Triton X‐100; 600 mM β‐glycerophosphate; 1 mM NaF; 0.2 M PMSF; 10 mg/mL LEU (L2884, Sigma‐Aldrich); and 1 M Na_3_VO_4_; pH 8.5) for 20 s. Total protein concentrations were determined using a DC protein assay kit (5000111, Bio‐Rad Laboratories). Equivalent amounts of protein (30 μg) from each sample were dissolved in 2× SDS sample buffer (20% glycerol; 100 mM Tris, pH 6.8; 4% SDS; 0.17% bromophenol blue; 0.25 M DTT) and heated at 95°C for 5 min. Samples were subjected to electrophoretic separation on 7.5–12% SDS‐PAGE acrylamide gels (Goodman et al., 2011). Proteins were then transferred to a methanol‐activated polyvinylidene difluoride (PVDF) membrane, blocked with 5% powdered skim milk in 0.1% TBS‐T (Tris‐buffered saline with 0.1% Tween 20) for 1 h, followed by an overnight incubation at 4°C with the appropriate primary antibody (Table 1 for antibody details) diluted in 0.1% TBS‐T containing 1% bovine serum albumin (BSA; A7030, Sigma‐Aldrich). Membranes were then washed for 30 min in 0.1% TBS‐T and then probed with a peroxidase‐conjugated secondary antibody (Table 1 for antibody details) for 1 h at room temperature. Membranes were then developed using enhanced chemiluminescence (ECL) plus reagent (1705061, Bio‐Rad Laboratories) and imaged with a Fusion‐FX7 imaging station (Vilber Lourmat, France). Densitometric measurements were taken using the Fusion‐CAPT software (Vilber Lourmat, France) for the proteins of interest. Once appropriate images were captured, the membranes were stained with Coomassie Blue to verify equal loading of total protein in all lanes. To account for minor variations in protein loading between lanes, protein bands of interest were normalized to the Coomassie Blue signal of the lane for total protein using ImageJ software (NIH, Bethsda, MD, USA).

**TABLE 1:** List of antibodies.

	Supplier	Catalog #	Dilution
**Primary antibody**			
APE1/Ref‐1 antibody mouse monoclonal	Novus Biologicals	13B8E5C2	1:1000
NRF2 antibody rabbit monoclonal	CST	12,721	1:1000
KEAP‐1 antibody rabbit monoclonal	CST	8047	1:1000
NF‐κB p65 antibody rabbit monoclonal	CST	8242	1:1000
Phosphorylated NF‐κB (pNF‐κB) p65 (Ser536) antibody rabbit monoclonal	CST	3033	1:1000
**Secondary antibody**			
Anti‐rabbit HRP‐conjugated IgG (H + L)	Vector Labs	PI‐1000	1:5000
Anti‐mouse HRP‐conjugated IgG (H + L)	Vector Labs	PI‐2000	1:20,000

### 2.7 Statistical analysis

All values are expressed as means ± SEM in graphs unless stated otherwise. A Shapiro–Wilk test was performed to assess normality. An unpaired *t*‐test was used to evaluate differences in APE1/Ref‐1 protein expression between strains, and differences in CD68 and CD45‐positive cells between treated and untreated *mdx* mice. In every other instance, four group comparisons were analyzed using a two‐way ANOVA with strain and treatment as the factors, followed by a Tukey post hoc analysis where there was a significant interaction. When data were not normally distributed, a Kruskal–Wallis test was performed for four‐group comparisons, followed by a Dunn post hoc analysis. Differences between groups were considered significant if *p* ≤ 0.05. All data analysis were performed using GraphPad Prism 9.0 (GraphPad Software Inc., La Jolla, CA, USA).

## 3 RESULTS

### 3.1 APE1/Ref‐1 is expressed in murine skeletal muscle

**FIGURE 1:**
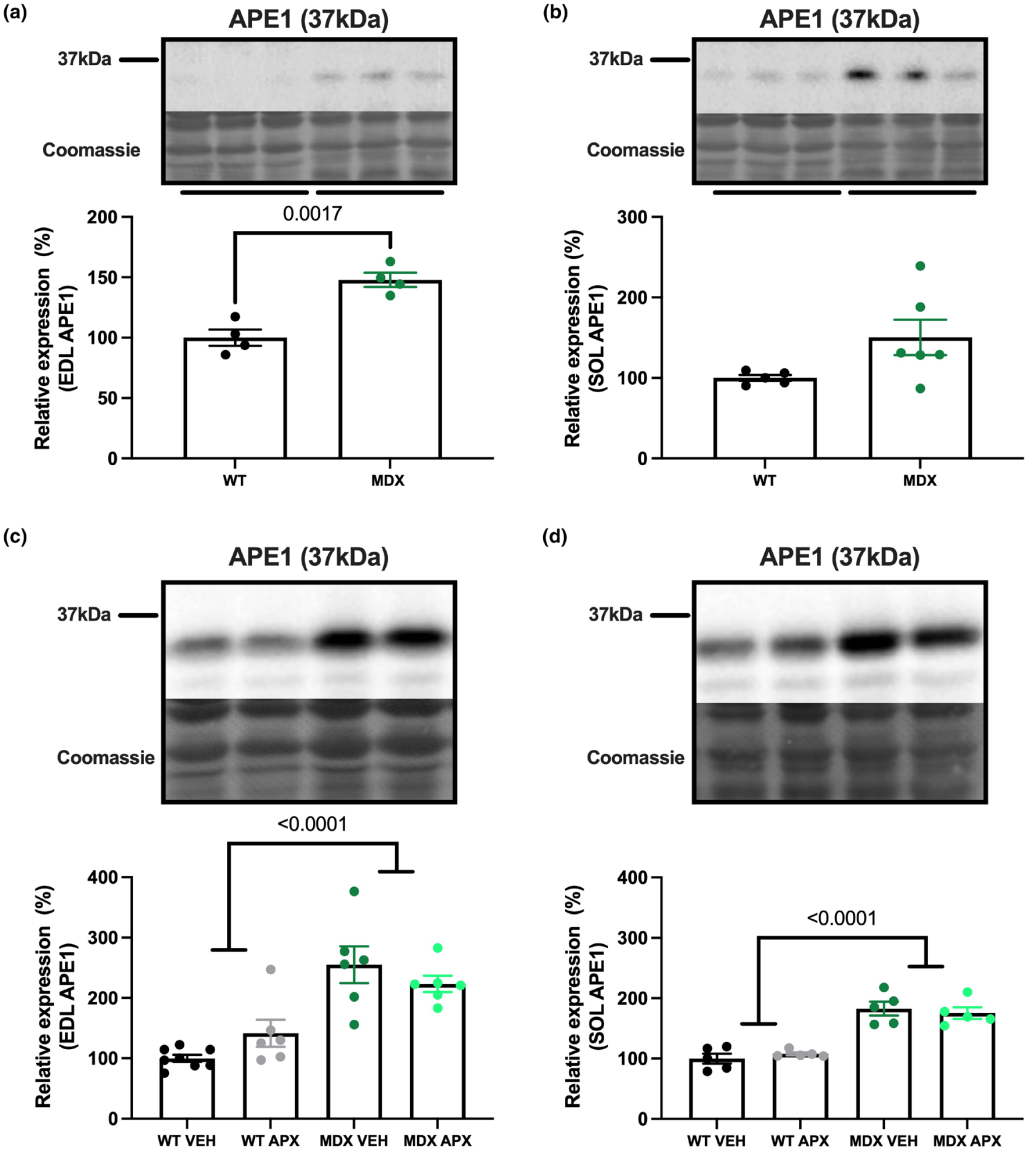
The protein expression of APE1‐Ref1 in wildtype (WT) and mdx (MDX) mice in skeletal muscle. APE1/Ref‐1 expression was initially analyzed in (a) EDL and (b) soleus muscle homogenates. The effect of APX3330 treatment on APE1/Ref‐1 expression was analyzed in the (c) EDL and (d) SOL. Statistically significant pairwise and four‐group comparisons are shown on the graphs. *n* = 4–6. APX, APX3330 treated; EDL, extensor digitorum longus; MDX, *mdx* mice; SOL, soleus; VEH, vehicle treated; WT, wild type mice.

Using a commercially available antibody that has been validated with APE/Ref‐1 knockout samples, ~36 kDa APE1/Ref‐1 was found to be expressed in fast‐ and slow‐twitch skeletal muscles, with elevated protein expression observed in dystrophic compared to wild type muscles (EDL *p* < 0.01; soleus *p* = 0.07; Figure 1a,b). APX3330 treatment had no significant effect on APE1/Ref‐1 expression in either *mdx* or wild type muscles (Figure 1c,d).

### 3.2 APX3330 does not improve the ex vivo contractile function of *mdx* hindlimb muscles

An APX3330 dose of 25 mg·kg^−1^ was used in accordance with previous studies in models of pancreatic cancer and ulcerative colitis, which was sufficient to lower inflammatory cell infiltration and activation of inflammatory pathways (Fishel et al., 2011; Sahakian et al., 2020).

As expected, EDL and soleus muscles from *mdx* mice were heavier than WT mice (*p* < 0.001 and *p* < 0.0001, respectively; Table 2) (Kornegay et al., 2012). Body weights were also higher in *mdx* mice than WT mice, and when normalized to body weight, there was no difference in EDL and soleus muscle masses between strains. APX3330 had no significant effect on absolute or relative muscle mass in either *mdx* or WT mice.

**TABLE 2:** Body mass and muscle morphology characteristics.

Measure	WT VEH *n* = 8		WT APX *n* = 8		*mdx* VEH *n* = 8		*mdx* APX *n* = 8	
Body mass (g)	22.2 ± 1.1		22.8 ± 0.9		23.3 ± 1.1		23.8 ± 0.9	
**Muscle measure**	**EDL**				**SOL**			
	**WT VEH *n = 8***	**WT APX *n = 8***	***mdx* VEH *n = 8***	***mdx* APX *n = 8***	**WT VEH *n = 8***	**WT APX** ***n = 8***	***mdx* VEH *n = 8***	***mdx* APX *n = 8***
Mass (mg)	9.2 ± 1.0	8.9 ± 0.5	10.9 ± 1.9*	11.8 ± 2.0*	7.3 ± 0.6	7.1 ± 1.1	9.7 ± 1.2*	9.2 ± 1.7*
*L*_0_ (mm)	13.0 ± 0.7	12.4 ± 0.4	12.3 ± 0.8	12.7 ± 0.5	11.7 ± 0.5	11.1 ± 0.3	11.3 ± 0.6	11.2 ± 0.4
*P*_*t*_ (mN)	36.2 ± 7.3	49.5 ± 6.7	43.2 ± 13.2	45.5 ± 14.4	20.4 ± 8.8^	24.7 ± 5.1	20.9 ± 3.3	21.4 ± 2.8
TTP (ms)	9.0 ± 0.4	9.1 ± 0.4	9.1 ± 0.5	9.0 ± 0.4	12.9 ± 0.4^	12.4 ± 1.0	12.9 ± 0.8	12.8 ± 0.7
½ RT (ms)	12.6 ± 0.3	13.4 ± 0.2	13.0 ± 0.2	12.5 ± 0.3	26.6 ± 0.4^	24.5 ± 0.5	25.6 ± 0.2	26.0 ± 0.3
*P*_*t*_/*P*_0_	0.14 ± 0.04	0.18 ± 0.02	0.19 ± 0.03*	0.19 ± 0.03*	0.14 ± 0.02^	0.14 ± 0.04	0.14 ± 0.02	0.14 ± 0.02
CSA (mm^2^)	0.15 ± 0.01	0.15 ± 0.01	0.19 ± 0.03*	0.20 ± 0.04*	0.08 ± 0.01	0.09 ± 0.01	0.11 ± 0.01*	0.11 ± 0.02*

*Note*: Symbols indicate: ^*n* = 7; *p ≤ 0.05, significantly different from WT VEH group using a two‐way ANOVA or Kruskal–Wallis.

Abbreviations: ½ RT, half relaxation time; APX, APX3330 treated; CSA, cross‐sectional area; EDL, extensor digitorum longus; L_0_, optimal length; MDX, *mdx* mice; P_0_, peak tetanic absolute force; P_t_, peak twitch; SOL, soleus; TTP, time to peak; VEH, vehicle treated; WT, wild type mice.

**FIGURE 2:**
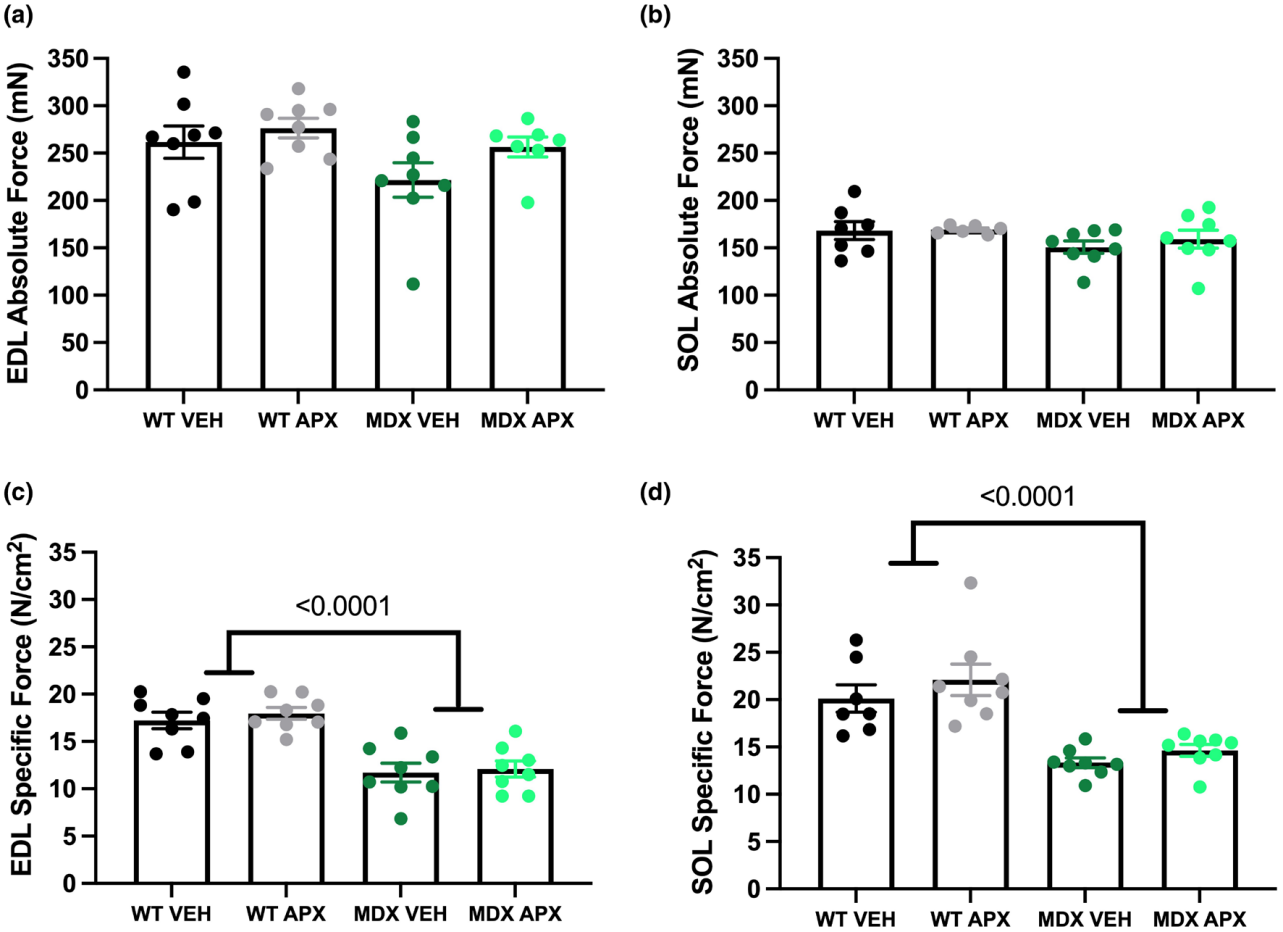
EDL and soleus absolute and specific force production in wild type and *mdx* mice. Absolute force of (a) EDL and (b) SOL. Specific force calculated as force produced per CSA of (c) EDL and (d) SOL. Statistically significant four‐group comparisons are shown on the graphs. *n* = 7–8. APX, APX3330 treated; EDL, extensor digitorum longus; MDX, *mdx* mice; SOL, soleus; VEH, vehicle treated; WT, wild type mice.

Optimal length (L_0_), peak twitch (P_t_), time to peak (TTP), and half relaxation time (½ RT) were neither significantly different between WT and *mdx* mice in either EDL or soleus muscles, nor were these twitch parameters affected by APX3330 treatment (Table 2). Consistent with higher absolute muscle mass, muscle cross‐sectional area (CSA) was significantly greater in EDL and soleus *mdx* mice (*p* < 0.0001; Table 2). In EDL and soleus muscle, peak absolute force (P_0_) did not differ between strains (Figure 2a,b); whereas, in concordance with the known dystrophic pathology of *mdx* mice (Coulton et al., 1988), the specific force (force per CSA; sP_0_) was lower in both EDL and soleus muscles from *mdx* mice compared to WT mice (*p* < 0.0001; Figure 2c,d). APX3330 treatment had no significant effect on the P_0_ and sP_0_ of EDL and soleus muscles from either *mdx* or WT mice.

**FIGURE 3:**
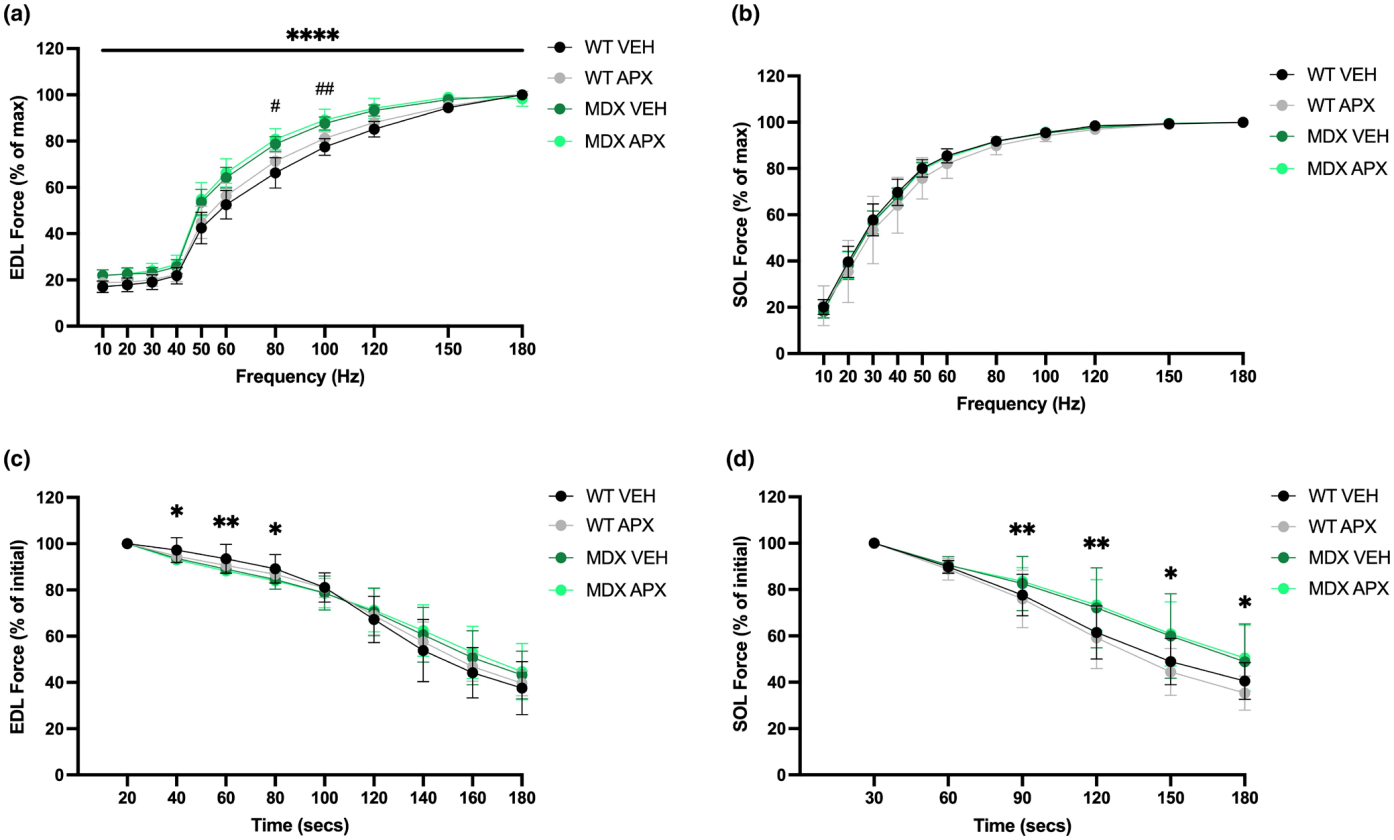
Force–frequency relationship and fatigue of EDL and soleus (SOL) in wild type and *mdx* mice. FFC of (a) EDL and (b) SOL. Fatigue of (c) EDL and (d) SOL. *p ≤ 0.05, **p ≤ 0.01, and ****p ≤ 0.0001 strain effect; ^#^ *p* ≤ 0.05 and ^##^ *p* ≤ 0.01 treatment effect; analyzed using a two‐way ANOVA. *n* = 8 per group. APX, APX3330 treated; EDL, extensor digitorum longus; FFC, force–frequency curve; MDX, *mdx* mice; SOL, soleus; VEH, vehicle treated; WT, wild type mice.

When muscle force at submaximal stimulation frequencies was normalized to maximal force output, there was a strain difference observed for the EDL, but not the soleus (*p* < 0.001; Figure 3a,b). Specifically, there was a leftward shift of the *mdx* force frequency curve, with the *mdx* EDL muscles producing a greater force at lower frequencies compared to the WT EDL muscles. Given there were no changes in twitch characteristics, this is likely due to a higher proportion of slow and/or regenerating fibers in the *mdx* EDL (Gregorevic et al., 2004; Webster et al., 1988). Contractile characteristics of muscle fibers in the early stages of regeneration are similar between fast and slow muscles; thus, in fast twitch muscles, recently regenerated fibers display a slower phenotype (Gregorevic et al., 2004). APX3330‐treated groups generated significantly larger force between 80 Hz and 100 Hz in the EDL, but no significant effect on the normalized force–frequency relationship of the soleus muscle (Figure 3a,b).

Strain differences in fatiguability were observed in the EDL muscles between *mdx* and WT mice between 40 s and 80 s, as *mdx* mice were more fatigable than WT mice (*p* < 0.05; Figure 3c,d). In contrast, soleus muscles from *mdx* mice were less fatigable than the WT soleus muscles between 90 s and 180 s of stimulation (*p* < 0.05; Figure 3d). APX3330 had no effect on EDL or soleus muscle fatiguability.

### 3.3 APX3330 alters the histomorphometry of hindlimb muscles

**FIGURE 4:**
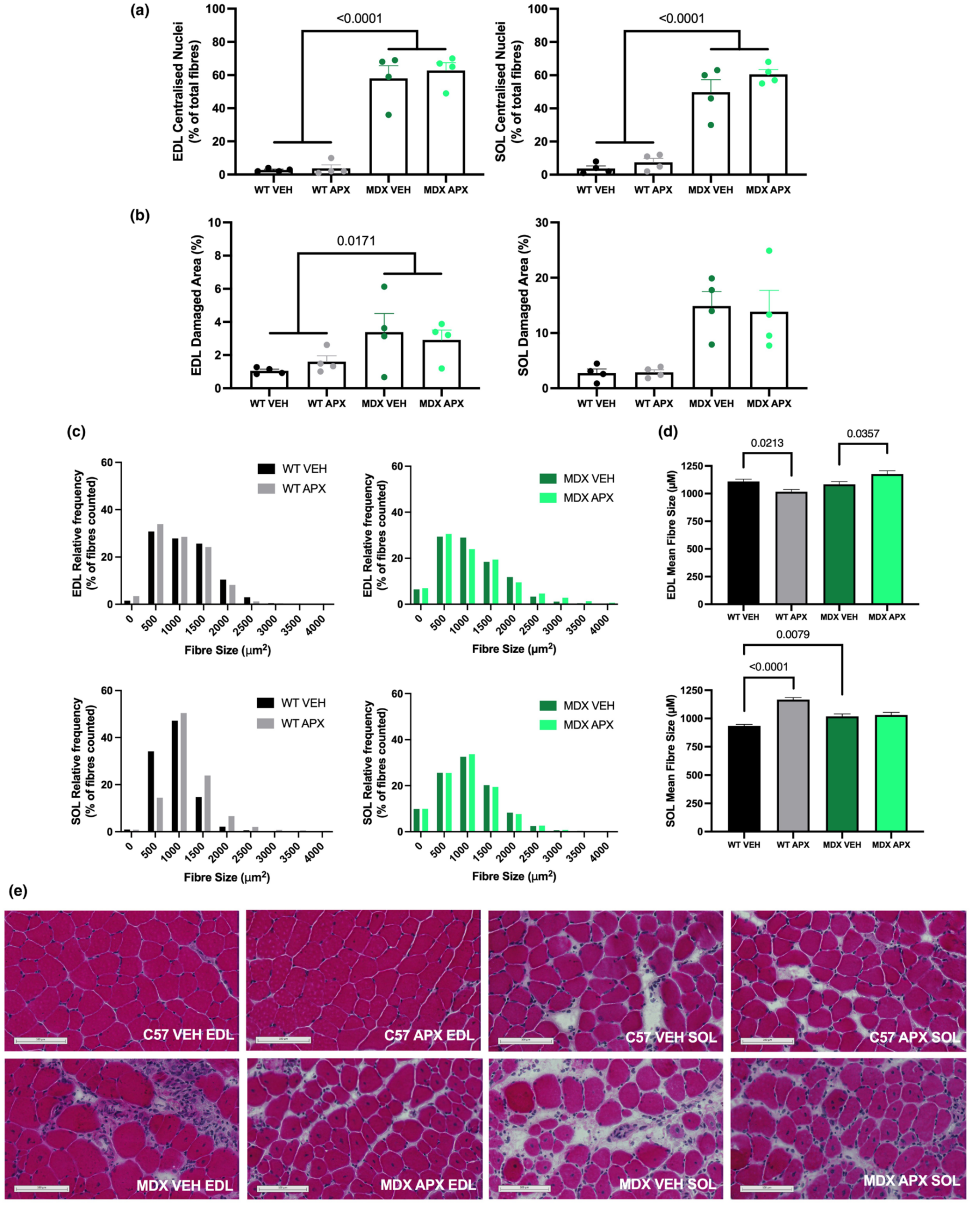
H&E histological analysis of the EDL and soleus in wild type and *mdx* mice. (a) Centralized nuclei displayed as a percentage of total fibers counted. (b) Damaged area as a percentage of the total area in the EDL and soleus. (c) Fiber size distribution in EDL and soleus. (d) Mean fiber size of EDL and soleus. Representative images are depicted in panel (e). Statistically significant four‐group comparisons are shown on the graphs. *n* = 4 per group. Scale bar = 100 μm. APX, APX3330 treated; EDL, extensor digitorum longus; MDX, *mdx* mice; SOL, soleus; VEH, vehicle treated; WT, wild type mice.

Typical of the dystrophic pathology, EDL and soleus muscles from *mdx* mice displayed a large proportion of recently damaged and regenerated fibers (characterized as centrally nucleated fibers) when compared to healthy WT muscle (*p* < 0.0001; Figure 4a); however, the proportion of centrally nucleated fibers was unchanged with APX3330 treatment. Muscle fiber damage analysis indicated that approximately 3% of the EDL *mdx* muscle area was damaged, and 10% of the soleus when compared to healthy WT (EDL *p* < 0.05; soleus *p* < 0.001), with no effect of APX3330 treatment (Figure 4b).

APX3330 treatment resulted in a slight leftward shift in the fiber area histogram in the EDL muscles of WT mice, with an overall 8% smaller fiber size (*p* < 0.001; Figure 4c). Interestingly, fiber size in the soleus of WT mice was 20% higher in the APX3330‐treated groups (*p* < 0.0001) (Figure 4d). There was an increase in mean fiber size (*p* < 0.05), with no change in distribution in *mdx* APX3330 mice (Figure 4c,d). The changes in fiber CSA did not translate to changes in contractile function.

**FIGURE 5:**
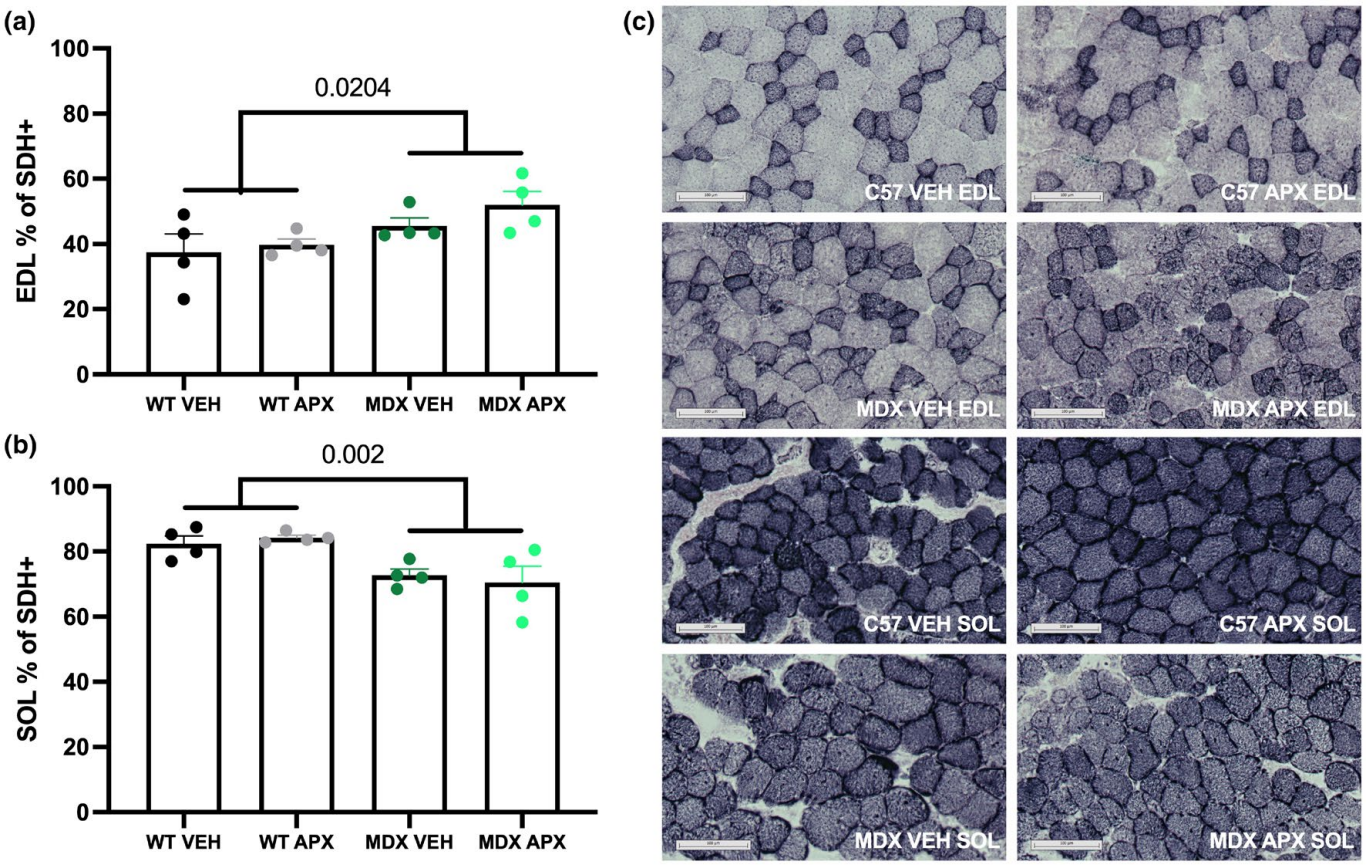
SDH histological analysis of the EDL and soleus in wild type and *mdx* mice. (a) SDH‐positive fibers as a percentage of total area in the EDL. (b) SDH‐positive fibers as a percentage of total area in the soleus. Representative images are depicted in panel (c). Statistically significant four‐group comparisons are shown on the graphs. *n* = 4 per group. Scale bar = 100 μm. APX, APX3330 treated; EDL, extensor digitorum longus; MDX, *mdx* mice; SOL, soleus; VEH, vehicle treated; WT, wild type mice.

Succinate dehydrogenase (SDH) stain analysis of oxidative and non‐oxidative muscle fibers showed no significant effect of APX3330 in either *mdx* or WT mice in both EDL and soleus muscles (Figure 5). This is consistent with the fatiguability results, where APX3330 had no significant effect. However, there was an overall strain effect, noting a higher oxidative capacity in the *mdx* EDL compared to the WT EDL (*p* < 0.05; Figure 5a), consistent with a slower, more oxidative phenotype. Despite higher oxidative capacity, as the overall oxidative capacity of the EDL is low, it was not enough to alter fatiguability. In contrast, a lower oxidative capacity in the *mdx* soleus compared to the WT muscles was observed (*p* < 0.01) (Figure 5b).

### 3.4 APX3330 lowers monocyte and macrophage infiltration but not leukocyte infiltration in the EDL

**FIGURE 6:**
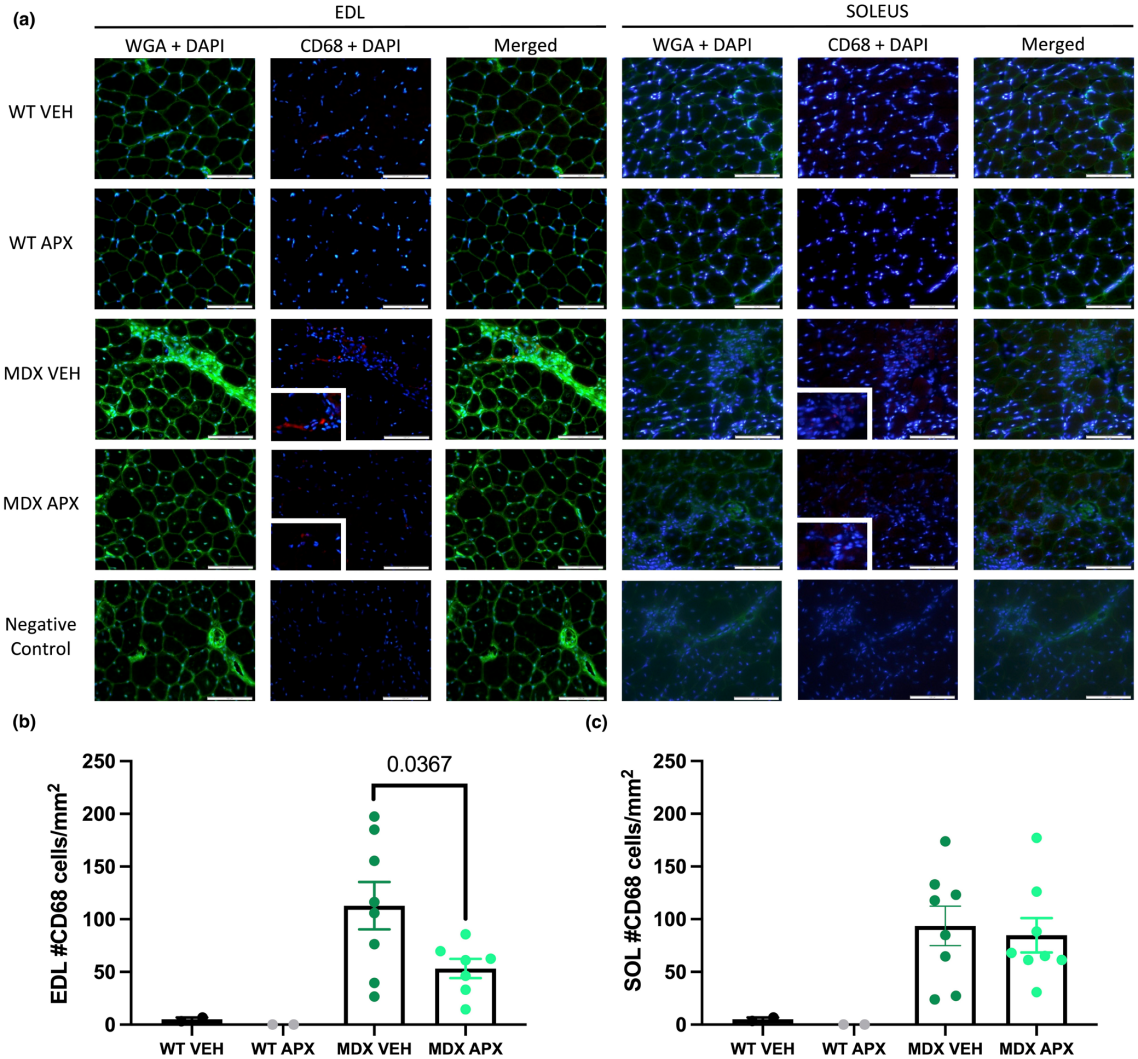
Monocyte and macrophage infiltration in the EDL and soleus of wild type and *mdx* mice. (a) CD68+ monocytes and macrophages were labeled using anti‐CD68+ (red) antibody in EDL and soleus cross‐sections. Connective tissue to visualize muscle architecture is labeled with WGA (green) and nuclei are labeled with nuclei marker DAPI (blue). (b) Quantification of CD68+ cells in EDL cross‐sections. (c) Quantification of CD68+ cells in soleus cross‐sections. Scale bar = 100 μm, 40× magnification. Statistically significant pairwise comparisons are shown on the graphs. *n* = 2 mice for wild type groups and *n* = 8 for *mdx* groups. APX, APX3330 treated; EDL, extensor digitorum longus; MDX, *mdx* mice; SOL, soleus; VEH, vehicle treated; WT, wild type mice.

CD68‐positive monocyte and macrophage infiltration were evident in the EDL muscles from *mdx* mice and was significantly lower in *mdx* mice treated with APX3330 (*p* < 0.05) (Figure 6a,b); however, this was not observed in the soleus muscle of *mdx* mice (*p* < 0.01) (Figure 6a,c).

CD45‐positive cells were also analyzed to compare the effect of APX3330 found here to those of other studies (Sahakian et al., 2020). CD45‐positive cells were also more abundant in *mdx* mice, indicative of an increase in leukocyte infiltration, although not to the same extent as CD68‐positive cells. Treatment with APX3330 did not significantly lower the level of CD45‐positive cells in *mdx* muscles.

Wild type groups were used to demonstrate that no infiltration of monocytes and macrophages occurs in healthy muscle and that APX3330 does not alter this in the wild type muscle due to the lack of inflammation occurring.

### 3.5 APX3330 altered inflammatory cell signaling pathways, not oxidative pathways, in hindlimb muscles

**FIGURE 7:**
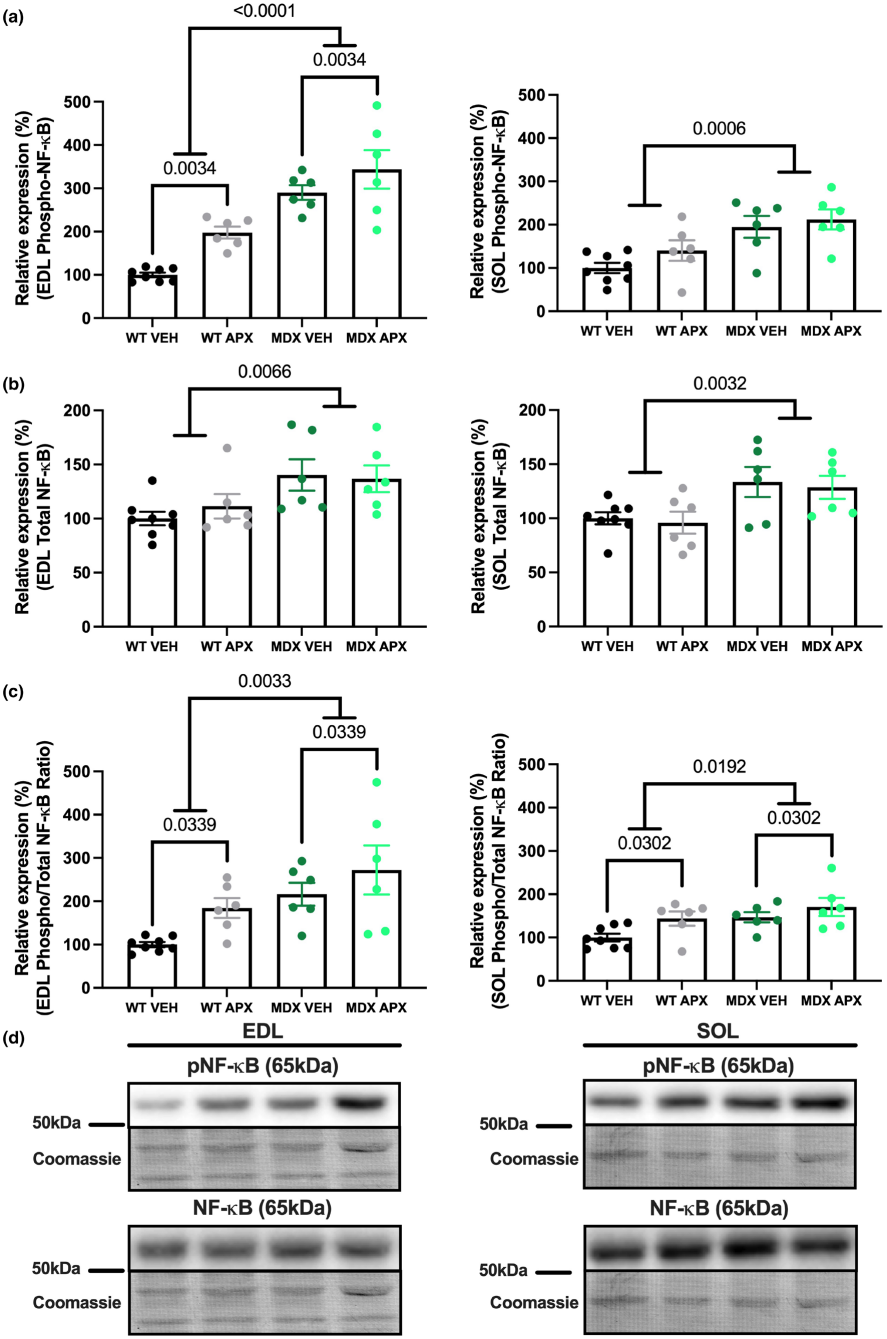
The effect of APX3330 on the protein expression of inflammatory markers in wild type and *mdx* mice. Western blotting experiments were undertaken on EDL and soleus muscle homogenates. Samples were probed for (a) phosphorylated NF‐κB p65 (Ser536) and (b) total NF‐κB. (c) The ratio of phosphorylated NF‐κB and total NF‐κB was utilized to indicate the activation of phosphorylated NF‐κB independent of the total amount of NF‐κB. Western blotting data are expressed as a relative percentage to the WT VEH group, and (d) representative images are displayed with Coomassie Blue representative image below, which was used as the protein loading control. Statistically significant four‐group comparisons are shown on the graphs. *n* = 6–8. APX, APX3330 treated; EDL, extensor digitorum longus; MDX, *mdx* mice; NF‐κB, nuclear factor kappa‐light‐chain‐enhancer of activated B cells; SOL, soleus; VEH, vehicle treated; WT, wild type mice.

**FIGURE 8:**
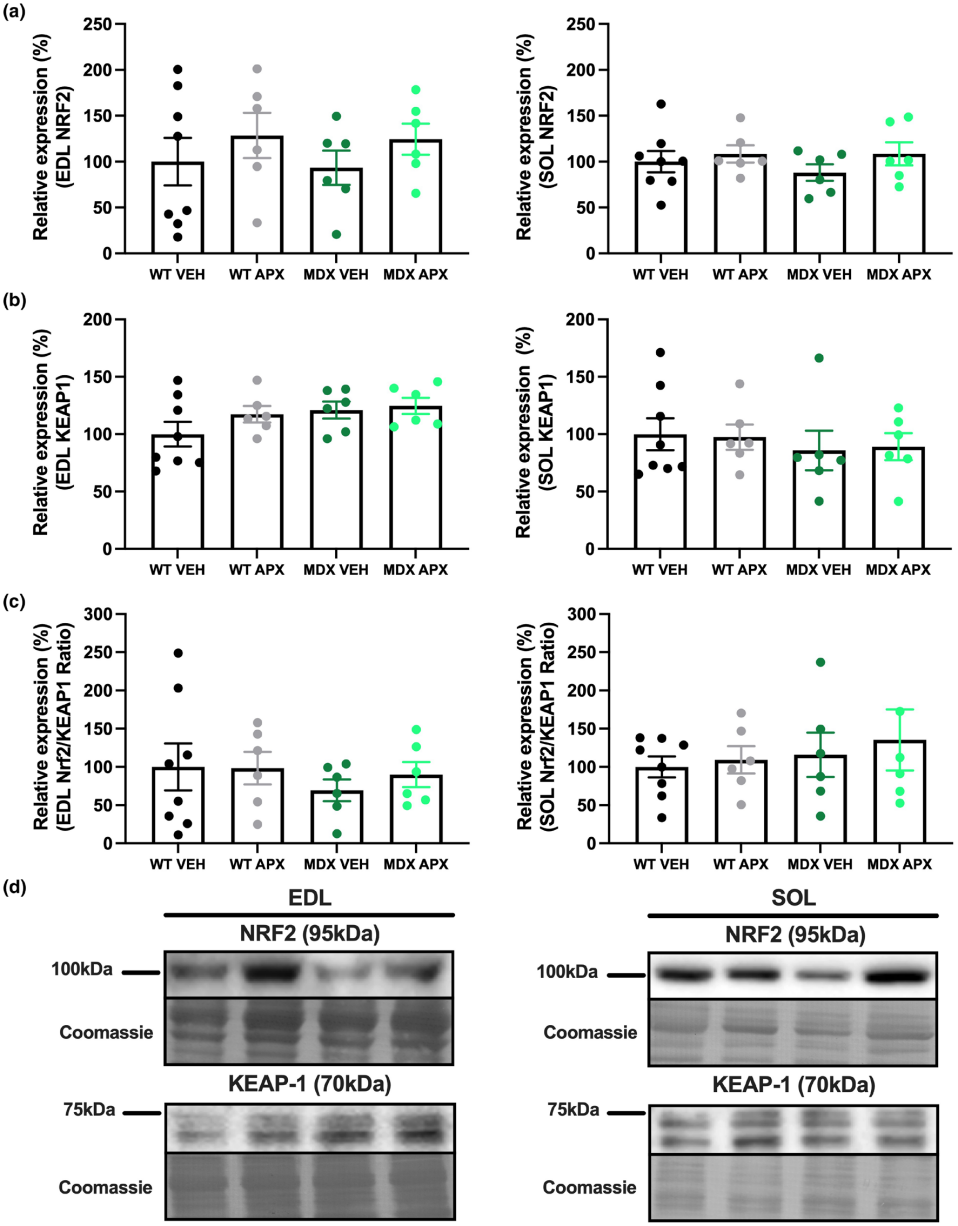
The effect of APX3330 on the protein expression of antioxidant markers in wild type and *mdx* mice. Western blotting experiments were undertaken on EDL and soleus muscle homogenates. Samples were probed for (a) NRF2 and (b) KEAP‐1. (c) The ratio of NRF2 and KEAP‐1 was utilized to indicate the activation of NRF2 activity independent of KEAP‐1. Western blotting data are expressed as a relative percentage to the WT VEH group and (d) representative images are displayed with Coomassie Blue representative image below, which was used as the protein loading control. *n* = 6–8. APX, APX3330 treated; EDL, extensor digitorum longus; KEAP1, Kelch‐like ECH‐associated protein 1; MDX, *mdx* mice; NRF2, nuclear factor erythroid 2‐related factor 2; SOL, soleus; VEH, vehicle treated; WT, wild type mice.

NF‐κB p65 phosphorylation leads to a pro‐inflammatory response; thus, the ratio of p‐NF‐κB p65 and total NF‐κB p65 is a typical indicator of increased inflammation (Christian et al., 2016). p‐NF‐κB, total NF‐κB, and the p‐NF‐κB/NF‐κB ratio were significantly higher in *mdx* muscles in comparison with WT mice (EDL *p* < 0.0001 and *p* < 0.01; soleus *p* < 0.001 and *p* < 0.01, respectively; Figure 7). Irrespective of strain, p‐NF‐κB in the EDL (*p* < 0.01) and the ratio of phosphorylated and total NF‐κB in both the EDL and soleus muscles from *mdx* and WT mice were higher with APX3330 treatment (*p* < 0.05). This was an unexpected observation given the lower number of CD68 monocytes and macrophages in EDL muscles from *mdx* mice and may demonstrate a disconnect between inflammatory cell infiltration and NF‐κB signaling. There were no significant differences in the expression of NRF2 or Kelch‐like ECH‐associated protein 1 (KEAP1) between any of the groups in either EDL or soleus muscles (Figure 8). As NRF2 is an activator of antioxidant pathways and KEAP‐1 inhibits NRF2 activation, the ratio between the two indicates the level of activated NRF2 (Motohashi & Yamamoto, 2004). It is important to note that NRF2 and KEAP‐1 protein expression were not significantly different between *mdx* and WT mice irrespective of muscle type, despite other indications of increased oxidative stress in *mdx* mice.

## 4 DISCUSSION

For the first time in skeletal muscle, we demonstrate that the APE1/Ref‐1 protein is expressed in fast and slow hindlimb muscles and, in concordance with its role in inflammation and oxidative stress, is elevated in hindlimb muscle from dystrophin‐deficient *mdx* muscles. The higher abundance of APE1/Ref‐1 protein in dystrophic EDL and soleus muscles is consistent with the low‐grade inflammation observed in hindlimb muscles from 12‐week‐old *mdx* mice and suggests that APE1/Ref‐1 may play a role in dystrophic pathology. Despite the elevation of APE1/Ref‐1 in hindlimb muscles from *mdx* mice, treatment with the APE1/Ref‐1 redox inhibitor, APX3330, did not alter the protein content of APE1/Ref‐1 protein, nor the expression of oxidative stress signaling proteins, NRF2 and KEAP‐1. Interestingly, phosphorylated NF‐κB was higher with APX3330 treatment. In *mdx* and WT mice, APX3330 had no significant effect on the strength and endurance of isolated EDL and soleus muscles. This does not appear to be due to the availability, as APX3330 has been shown to enter skeletal muscle in tracer studies within 15 min of an oral dose, remaining elevated 2 h later, and still detectable 48 h later (Professor Mark Kelley, Indiana University School of Medicine, personal communication). Indeed, APX3330 treatment in the current study did lower the number of CD68‐positive monocytes and macrophages, which is in agreement with its expected role as an anti‐inflammatory drug. However, this was insufficient to improve the ex vivo strength and endurance of dystrophic EDL and soleus muscles.

Irrespective of mouse strain or muscle phenotype, APE1/Ref‐1 expression was unchanged with the treatment of APX3330. This may be due to APX3330 only inhibiting the redox function of APE1/Ref‐1, without affecting the regulation of its transcription, translation, or stability (Zhang et al., 2013). Treatment with APX3330 did not show any significant benefit in the twitch properties, tetanic force, or fatigue characteristics or EDL and soleus muscles from *mdx* and WT mice, yet interesting effects on fiber size were observed. Specifically, with APX3330 treatment, WT mice had smaller fibers in the EDL, but larger fibers in the soleus. The reason for these APX3330‐induced changes in muscle fiber size is currently unclear; however, these changes were not associated with changes in muscle force output. Clearly, these genotype‐ and muscle‐dependent effects of APX3330 on muscle fiber size require further investigation to elucidate the mechanism of action.

Low levels of muscle damage were observed in the EDL (3%) and soleus (10%), which is typical of the age of the *mdx* mice where inflammation and oxidative stress are stabilized at a moderately elevated state (Radley‐Crabb et al., 2014). Contrary to the long‐held view that fast‐twitch fibers are preferentially affected in dystrophic muscles (Karpati et al., 1988; Webster et al., 1988), and the fact that slow‐twitch fibers are more protected from contraction‐induced damage (Consolino & Brooks, 2004; Kiriaev et al., 2021), the soleus displayed more damage in comparison with the EDL. This may be due to the soleus exhibiting more degeneration at the beginning of ambulation/earlier stages of the disease, as the soleus is more activated than the EDL during ambulation in a mouse (Carnwath & Shotton, 1987; Hu et al., 2017). Alternatively, the fast‐twitch fibers, predominant in the EDL, are more affected during the peak damage and repair phase, while the soleus undergoes a slower, more sustained, lower level of damage as the disease progresses, similar to human DMD. This is not unreasonable when the rough 40%–60% split of type I and II fibers (Augusto et al., 2004), respectively, in the soleus is more reflective of human muscle fiber composition, albeit the mouse soleus generally contains only type IIa fibers (Carroll et al., 2012).

Previously, APX3330 has been shown to decrease the abundance of inflammatory cells and factors in models of ischemia and colitis (Sahakian et al., 2020; Yan et al., 2018). In the Winnie mouse model of colitis, APX3330 reduced the amount of CD45‐positive leukocytes in the colon and the levels of inflammatory marker, lipocalin‐2 (Lcn‐2), in fecal pellets (Sahakian et al., 2020). In addition, APX3330 promoted the polarization of M2 macrophages in the ischemic brain of type 1 diabetes mellitus (T1DM) stroke rats, as indicated by a decrease in ED1, an M1 macrophage marker, and increases in CD163, an M2 macrophage marker (Yan et al., 2018). Yan et al. (2018) also noted decreases in inflammatory factors, including plasminogen activator inhibitor type 1 (PAI‐1), monocyte chemotactic protein‐1 (MCP‐1), and matrix metalloproteinase 9 (MMP9), after stroke in rats treated with APX3330 (Yan et al., 2018). CD45‐positive cells were analyzed in this study in accordance with previous studies yet showed no change with APX3330 treatment. Interestingly, APX3330 reduced CD68‐positive monocytes and macrophages in EDL muscles from *mdx* mice, with no significant difference in the soleus. As macrophages and monocytes in dystrophic muscles come primarily from the circulation, APE1/Ref‐1 may affect their adhesion to capillaries and migration from the circulation to damaged tissue (Petrof, 2022). Alternatively, APX3330 may be acting systemically, lowering the number of circulating monocytes and macrophages; therefore, limiting the number of CD68‐positive cells available to infiltrate skeletal muscle. Interestingly, the lowering of monocyte and macrophage infiltration did not ameliorate muscle pathology, particularly damaged area and function, suggesting that the extent of monocyte/macrophage infiltration is not tightly associated with the extent of the DMD pathology. This is consistent with our finding that treatment with APX3330 had no significant effect on percentage degeneration and centralized nuclei in EDL and soleus muscle cross sections, nor did it improve force output.

There is increasing interest in regulating redox signaling in DMD, with NRF2 activation and NF‐κB inhibition identified as potential therapeutic targets (Kourakis et al., 2021; Messina et al., 2011). NRF2 regulates the expression of many antioxidant and detoxification enzymes in response to oxidative stress (Ma, 2013). Under normal conditions, NRF2 is sequestered to the cytoplasm by forming a complex with the E3 ubiquitin ligase, KEAP‐1, which promotes NRF2's ubiquitination and degradation (Motohashi & Yamamoto, 2004; Petrillo et al., 2017). In response to an increase in reactive oxidation species (ROS), NRF2 is released from KEAP‐1 (Motohashi & Yamamoto, 2004) and dissociated NRF2 is transported to the nucleus to induce the transcription of antioxidant genes (Wardyn et al., 2015). In the current study, the expression of NRF2, KEAP‐1, and the NRF2/KEAP‐1 ratio was not significantly different between strains, nor altered with APX3330 treatment. This may indicate that NRF2 protein expression is not altered in DMD but, instead, may have impairments in the activation of its downstream effectors. Therefore, although APX3330 treatment did not influence protein expression of NRF2, investigation of downstream targets will be required to confirm.

The chronic activation of NF‐κB signaling heightens inflammation and compromises regeneration in dystrophic muscle (Proto et al., 2013). As expected, p‐NF‐κB and total NF‐κB were higher in EDL and soleus muscles from *mdx* mice compared to WT mice (Acharyya et al., 2007; Kumar & Boriek, 2003); however, unexpectedly, the abundance of p‐NF‐κB in the EDL and the ratio of phosphorylated and total NF‐κB in both the EDL and soleus muscles from both *mdx* and WT mice were higher with APX3330 treatment. This finding could suggest that APX3330 increased inflammatory signaling; however, the phosphorylation of the p65 subunit at Ser^536^ may act as a negative regulator of NF‐κB and, therefore, inhibit its signaling to prevent deleterious effects of prolonged inflammation (Pradere et al., 2016). Specifically, Pradere et al. (2016) used a phosphorylation‐deficient S534A knock‐in mouse (murine homolog of human Ser^536^) to show that p65 S534 phosphorylation is not required for its nuclear translocation. In fact, the inability to phosphorylate S534 actually increased inflammation‐induced NF‐κB‐dependent gene expression, in part, due to increased stability of the p65 subunit (Pradere et al., 2016). Thus, the higher abundance of p‐NF‐κB found in EDL muscles may be indicative of negative regulation of the NF‐κB pro‐inflammatory pathway, which correlates with the lower number of infiltrating CD68‐positive cells. Therefore, the higher p‐NF‐κB p65 Ser^536^ abundance and lowered number of CD68‐positive cells may indicate that treatment with APX3330 may have lowered the inflammatory response in skeletal muscle; however, this requires further confirmation. Nonetheless, this would again suggest that there is a degree of dissociation between inflammatory response and the *mdx* pathology.

This study is not without its limitations. For example, as APE1/Ref‐1 regulates the redox‐dependent transcription factor binding to DNA, APX3330 interferes with this process to potentially inhibit gene expression. Thus, treatment with APX3330 may have more of a direct effect on the transcription of target genes rather than an indirect effect via the regulation of the protein content of NF‐κB and NRF2/KEAP‐1 (Cesaratto et al., 2013). In addition, although CD45 was unchanged with APX3330 treatment, the CD45 antibody stains T and B cells very strongly, while macrophages and neutrophils, which are stained more weakly (Stupka et al., 2000), yet in DMD muscles, the predominant inflammatory cell populations are macrophages and neutrophils. Thus, the CD45 marker would not be as relevant in this context (Rosenberg et al., 2015). Furthermore, a higher dose of APX3330 may be required to further inhibit inflammation and have a positive impact on muscle pathology and function in *mdx* mice. Thus, further dosage studies may be required to determine the efficacy of APX3330 in skeletal muscle and whether these changes are due to direct action in the muscle or indirect action via alternative pathways. Decreases in inflammation that do not lead to improvements in muscle physiology may indicate that solely targeting inflammation, as with the current standard of care, is insufficient in treating dystrophy and that a more multifaceted approach targeting multiple aspects of the pathology would be more beneficial. Thus, further studies are required to elucidate the role of APE1/Ref‐1 in dystrophic muscle either via the generation of muscle‐specific knock‐in/out models, or by implementing APX3330 at an earlier time point during periods of heightened damage and repair, offering the most ‘treatable’ age consistent with DMD being a pediatric disease. Additionally, APE1/Ref‐1 localization or inhibition of endonuclease activity of APE1/Ref‐1 were not assessed in this study and, thus, studies into these aspects may reveal further insight into the role of APE1/Ref‐1 and the effect of its inhibition.

This study is the first to show that APE1/Ref‐1 is not only expressed in skeletal muscles but also is elevated in DMD pathology. However, it is unclear whether it is upregulated to alleviate muscle damage or due to prolonged chronic inflammation. Nevertheless, this study demonstrates that APE1/Ref‐1 inhibition with APX3330 lowers monocyte/macrophage infiltration, yet this did not translate to meaningful changes in muscle structure and function. However, as this study focuses on a *mdx* pathology during a period of constant low‐grade damage (6–12 weeks of age), APX3330 treatment during a phase of heightened inflammation may yield more beneficial changes (3–6 weeks of age). Nonetheless, the current results may indicate the need for a treatment that targets multiple aspects of DMD pathology rather than inflammation alone.

## AUTHOR CONTRIBUTION

H.L: Investigation, data collection and analysis, writing—original draft, review, and editing. C.A.G: Conceptualization, supervision, and writing—review and editing. N.S: Formal analysis, visualization, and writing—review and editing. N.G: Data collection and writing—review and editing. D.A.D: Data collection and writing—review and editing. L.S: Providing APX3330, technical advice, and writing—review and editing. K.N: Providing APX3330, technical advice, and writing—review and editing. A.H: Conceptualization, supervision, and writing—review and editing.

## FUNDING INFORMATION

No funding information provided.

## CONFLICT OF INTEREST STATEMENT

The authors have no conflicts to declare.

## ETHICS STATEMENT

This study was approved by the Animal Ethics Committee at Victoria University (AEC 17/007). All experiments conformed to the Australian Code for the care and use of animals for scientific purposes (8th ed., 2013).

## Supporting information


Figure S1.

## Data Availability

The authors confirm that the data supporting the findings of this study are available within the article and/or its Supporting Information.
