# Supplementary material for: APE1/Ref‐1 inhibition via APX3330 lowers monocyte/macrophage infiltration without ameliorating the structure and function of dystrophic mdx hindlimb muscles
Source: Physiol Rep. 2025 Aug 1;13(15):e70494. doi: 10.14814/phy2.70494 (PMC12314421; doi:10.14814/phy2.70494)
Supplement: Supplementary file 1 — Figure S1. [file PHY2-13-e70494-s001.docx]

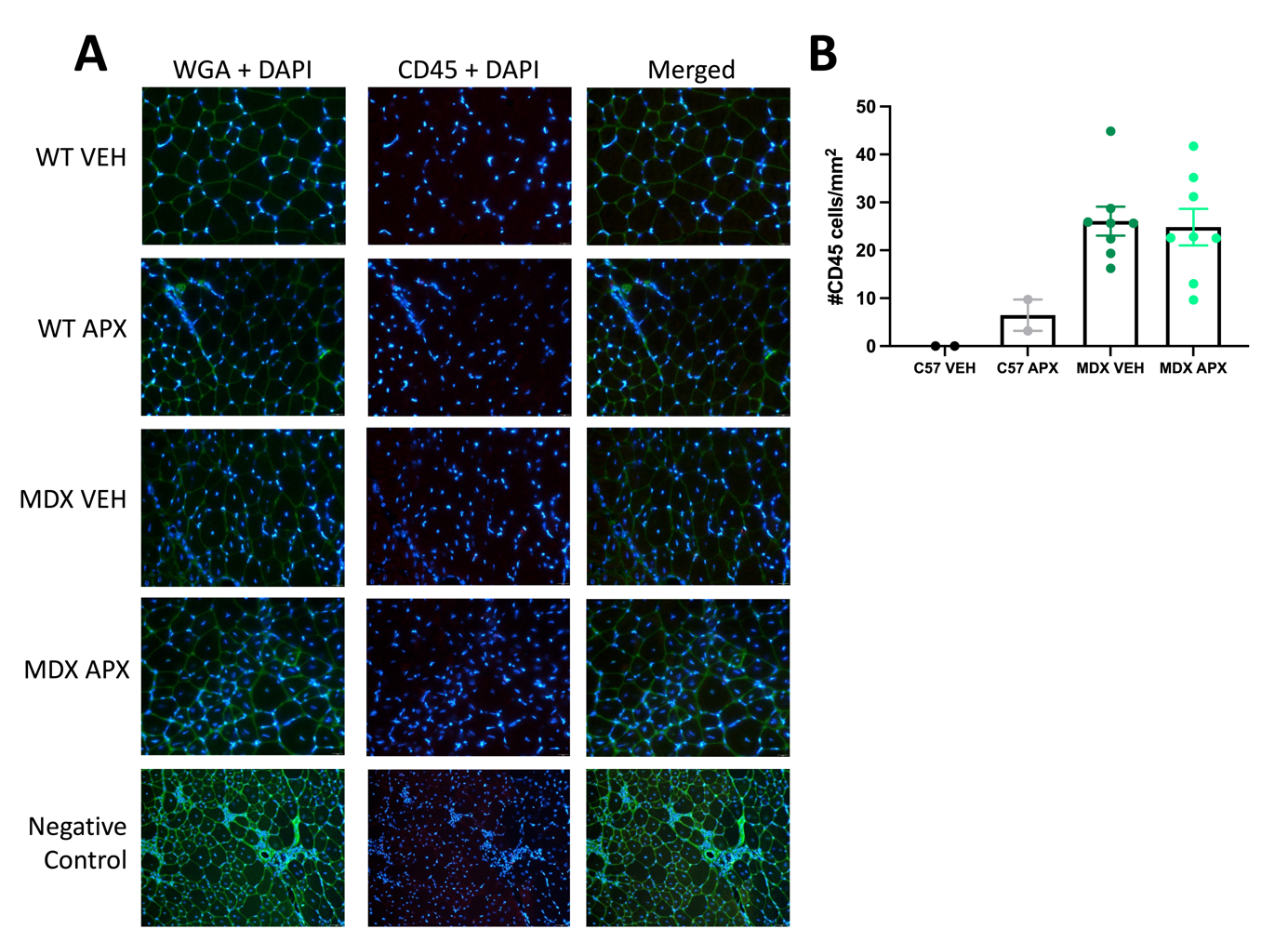


**Supplementary Figure A.** **Leukocyte infiltration in the EDL of wildtype and *mdx* mice.** **(A)** CD45+ leukocytes were labelled using leukocyte marker anti-CD45+ (red) antibody in EDL cross-sections. Connective tissue to visualize muscle architecture is labelled with WGA (green) and nuclei are labelled with nuclei marker DAPI (blue). **(B)** Quantification of CD45+ cells. Scale bar = 20µm, x40 magnification. n = 2 mice for wildtype groups and n = 8 for *mdx* groups. WT = wildtype mice; MDX = *mdx* mice; VEH = vehicle treated; APX = APX3330 treated; EDL = extensor digitorum longus.
